# A viability assay combining palladium compound treatment with quantitative PCR to detect viable* Mycobacterium avium* subsp*. paratuberculosis* cells

**DOI:** 10.1038/s41598-022-08634-x

**Published:** 2022-03-19

**Authors:** Martina Cechova, Monika Beinhauerova, Vladimir Babak, Petr Kralik

**Affiliations:** 1 Department of Microbiology and Antimicrobial Resistance, Veterinary Research Institute, Brno, Czech Republic; 2 Department of Experimental Biology, Faculty of Science, Masaryk University, Brno, Czech Republic; 3 Laboratory of Neurobiology and Pathological Physiology, Institute of Animal Physiology and Genetics, Czech Academy of Sciences, Libechov, Czech Republic

**Keywords:** Microbiology, Infectious-disease diagnostics

## Abstract

*Mycobacterium avium* subsp*. paratuberculosis* (MAP) is a pathogenic bacterium causing the paratuberculosis, chronic and infectious disease common particularly in wild and domestic ruminants. Currently, culture techniques to detect viable MAP are still used most commonly, although these require a long incubation period. Consequently, a faster molecular method for assessing MAP cell viability based on cell membrane integrity was introduced consisting of sample treatment with the intercalation dye propidium monoazide (PMA) followed by quantitative PCR (qPCR). However, the PMA-qPCR assay is complicated by demanding procedures involving work in a darkroom and on ice. In this study, we therefore optimized a viability assay combining sample treatment with palladium (Pd) compounds as an alternative viability marker to PMA, which does not require such laborious procedures, with subsequent qPCR. The optimized Pd-qPCR conditions consisting of 90 min exposure to 30 µM bis(benzonitrile)dichloropalladium(II) or 30 µM palladium(II)acetate at 5 °C and using ultrapure water as a resuspension medium resulted in differences in quantification cycle (Cq) values between treated live and dead MAP cells of 8.5 and 7.9, respectively, corresponding to approximately 2.5 log units. In addition, Pd-qPCR proved to be superior to PMA-qPCR in distinguishing between live and dead MAP cells. The Pd-qPCR viability assay thus has the potential to replace time-consuming culture methods and demanding PMA-qPCR in the detection and quantification of viable MAP cells with possible application in food, feed, clinical and environmental samples.

## Introduction

*Mycobacterium avium* subsp. *paratuberculosis* (MAP) is a slow-growing and highly resistant bacterium causing paratuberculosis, particularly in wild and domestic ruminants, e.g. deer, sheep, cattle and goats^1^. Clinical symptoms of this chronic and infectious disease appearing after a years-long latent period include diarrhea, reduced milk production, weight lost and exhaustion leading to the death of infected individuals^2^. The commonest sources of infection are feces and milk from MAP-infected animals^3^, though MAP can also be transmitted vertically to the fetus during an intrauterine infection^4^. MAP is also likely to participate in the development of Crohn’s disease in humans who may be exposed to it through contaminated milk, meat and water^5^.

Cultivation is one of the commonest methods used for the direct detection of viable MAP cells, though its disadvantages are the long incubation period (at least 12 weeks) and the need for preliminary chemical decontamination, which can reduce its sensitivity^6^. In addition, cultivation is not able to detect MAP cells present in a dormant or viable non-culturable state^1^, and the clumping of MAP cells increases the variability and decreases the accuracy of the data determined by this method^7^. In order to overcome these shortcomings, a molecular technique combining the treatment of the sample with the intercalating dye propidium monoazide (PMA) with quantitative PCR (qPCR) of the single-copy sequence *F57* (PMA-qPCR), has been introduced to assess the MAP viability^8^. The discrimination between viable and dead cells is based on membrane integrity. PMA dye penetrates selectively into the dead membrane-compromised cells, but not into viable cells with intact cell membranes. PMA molecules inside the cell intercalate into the DNA, whose modifications are irreversible after irradiation with visible light, which interferes with the subsequent amplification of the target sequence by qPCR. The advantages of PMA-qPCR over cultivation are time saving and higher sensitivity, although a demanding procedure requiring manipulation in a darkroom is a major limitation. In addition, bacterial suspensions need to be kept on ice during irradiation when a rise in temperature could disrupt the cell membranes of viable cells, into which the dye could subsequently penetrate^9^.

Platinum (Pt) and palladium (Pd) compounds proved to be suitable alternative viability markers to PMA dye, showing a similar potential to distinguish between viable and dead cells or infectious and non-infectious virus particles based on cell membrane integrity^10,11^ or capsid integrity^12,13^. In contrast to PMA, Pt and Pd compounds are not sensitive to visible light, therefore they do not require manipulation in a darkroom or on ice, their effect is not conditioned by excitation by light, and they are less expensive. Regarding comparison of these two metals, Pd compounds have been shown to be more convenient to use than Pt compounds as lower concentrations are needed for the viability assay, resulting in lower reagent costs^10^.

In the present study, we therefore aimed to optimize a viability assay combining Pd compound treatment with qPCR (Pd-qPCR) for selective detection of viable mycobacterial cells, as an alternative approach to Pt-qPCR addressed in a previous study^14^. First, we evaluated the effect of four Pd compounds on MAP DNA, selecting for the compound that binds the most to the target MAP sequence and prevents its amplification by qPCR. Subsequently, the most suitable concentrations, exposure time, temperature and resuspension medium were optimized for selected Pd compounds on *M. smegmatis*, as a fast-growing model mycobacterium with a continuation on the target slow-growing mycobacterium MAP. Finally, we compared the optimized Pd-qPCR protocol with the reference PMA-qPCR to reveal which viability assay allows more accurate distinction between viable and dead MAP cells.

## Methods

### Preparation of mycobacterial cultures

Mycobacterial cultures were prepared in the same way as in the previous study by Cechova et al.^14^. Fast-growing *M. smegmatis* (collection strain ATCC 700084) was used for initial optimization of Pd compound treatment, and slow-growing MAP (field isolate 7072) was subsequently exposed to the optimized conditions. Both mycobacterial strains were first grown on Herrold’s egg yolk medium (HEYM), with the addition of 2 µg/ml of Mycobactin J (Allied Monitor, USA) (HEYM-MJ) in the case of MAP, at 37 °C. The grown cultures were then inoculated into Middlebrook 7H9 broth (Difco, Livonia, USA) supplemented with Middlebrook OADC enrichment (Difco), and with the addition of 2 µg/ml of Mycobactin J for MAP, and incubated at 37 °C with shaking until an optical density at 600 nm (OD_600_) of about 1.0 (BioPhotometer; Eppendorf, Germany) was attained. The cultures were washed with ultrapure water (Top-Bio, Czech Republic), homogenized by vortexing with 1 mm zirconia beads (BioSpec, USA), and centrifuged at 100 × g for 30 s to eliminate big cell clumps. The upper portion of the bacterial suspension was diluted with ultrapure water to an OD_600_ of 0.15, which was determined as 10^7^ copies/ml by subsequent qPCR, and distributed in a volume of 500 µl per microtube.

In addition, the cultivation was used as a reference method for the quantification of viable *M. smegmatis* cells in the suspension, dispensing 100 µl of serial dilutions in triplicates on plates containing HEYM medium and incubating for 3 days at 37 °C until colony-forming units (CFU) were calculated.

### Preparation of dead mycobacterial cells

Dead cells were prepared in the same manner as in the previous study by Cechova et al.^14^ by placing 500 µl aliquots of bacterial suspension in a thermoblock tempered at 100 °C for 4 min (with shaking at 100 rpm) and then cooling them immediately on ice. To verify cell killing, heat-treated cell suspensions of *M. smegmatis* and MAP were seeded on HEYM and HEYM-MJ agar, respectively, and incubated at 37 °C for 2 weeks and 4 weeks, respectively. Cell rupture and DNA release during the heat treatment for the killed cells (*M. smegmatis* as well as MAP) was ruled out on the basis of the fact that no differences in Cq values were noticed between the heat-killed and non-heat-treated (both unexposed to Pd compounds) cells in qPCR.

### Preparation of palladium compounds and propidium monoazide

Four Pd compounds, previously reported in the study by Soejima and Iwatsuki^10^, were used to assess mycobacterial cell viability: dichloro(n-cycloocta-1,5-diene)palladium(II), bis(benzonitrile)dichloropalladium(II), palladium(II)acetate (Sigma-Aldrich, USA) and trans-diammine dichloropalladium(II) (Alfa Aesar, USA). The compounds were dissolved to the appropriate concentration (see below) in physiological saline solution with heating at 40 °C for approximately 1 h with shaking (1,000 rpm). PMA was prepared by the dissolution of 1 mg of PMA (Biotium, USA) in 1.9 ml of 20% dimethyl sulfoxide (DMSO; Sigma-Aldrich) to obtain a 1 mM solution^8^.

### The effect of palladium compounds on DNA amplification

**Figure 1:**
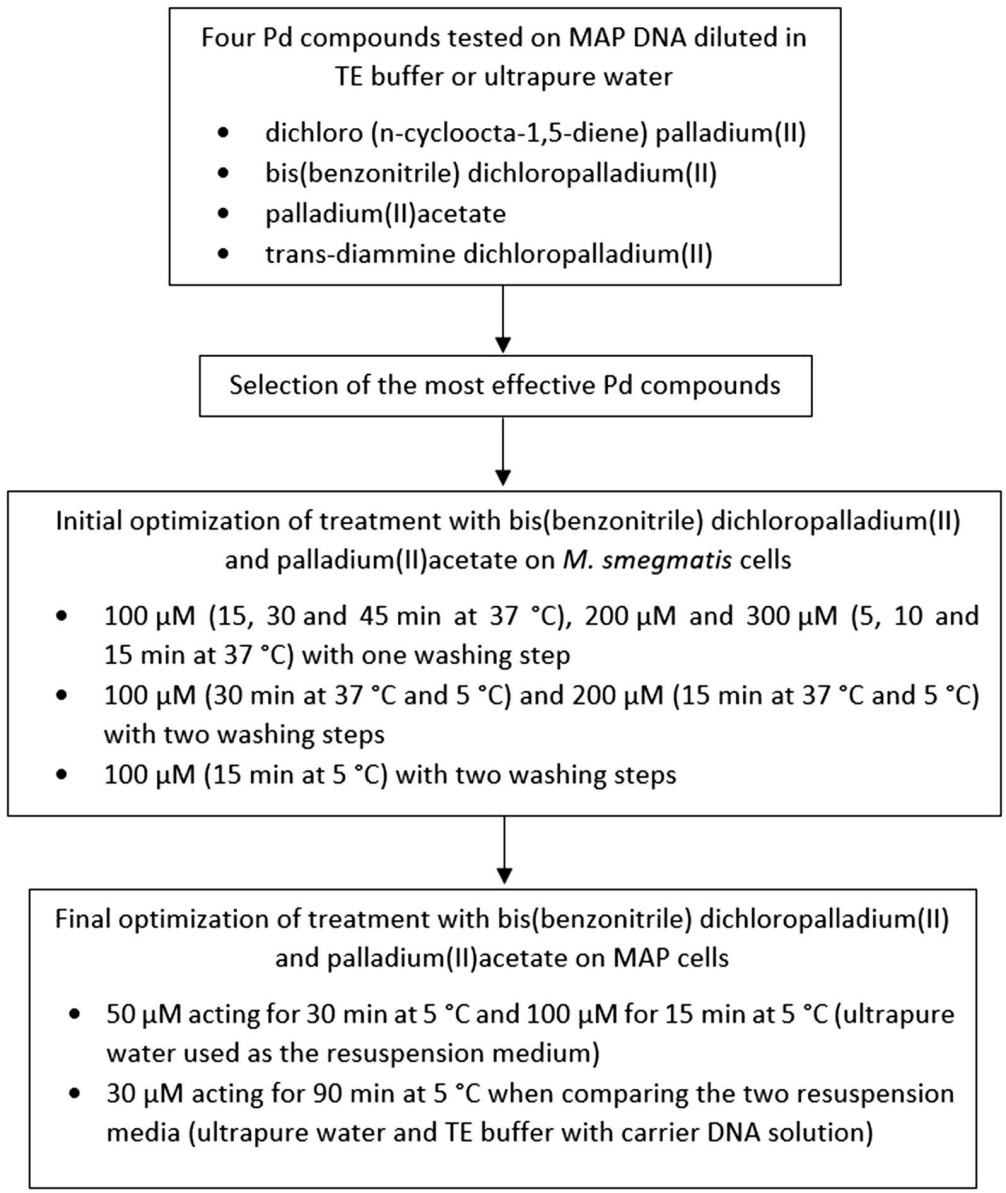
Schematic overview of the optimization of Pd compound treatment.

The same initial procedure as in the previous study^14^ was followed. An MAP cell suspension, prepared by resuspension of one loop of MAP culture in 200 µl of ultrapure water, was subjected to DNA isolation using a Quick-DNA Fecal/Soil Microbe Microprep kit (Zymo Research, Tustin, California, USA) according to the manufacturer’s protocol. Additionally, the original protocol was upgraded with the mechanical homogenization of MAP with 0.1 mm zirconia silica beads (Biospec) in a MagNA Lyser instrument (Roche Diagnostics GmbH, Mannheim, Germany) at 6,400 rpm for 1 min. The concentration of isolated DNA was determined using a NanoDrop 2000c spectrophotometer (Thermo Fisher Scientific, USA), and subsequently the DNA was divided into two parts, with the first diluted with ultrapure water and the second with tris-ethylenediaminetetraacetic acid (EDTA; TE) buffer (Serva Electrophoresis GmbH, Germany) to 1 ng/µl, and dispensed at 50 µl per microtube. Each Pd compound solution was added individually to the caps of the microtubes filled with the DNA solution, which were subsequently closed and uniformly spun to mix the Pd compounds and DNA solutions to reach a 10 µM, 100 µM and 1000 µM concentration. Incubation with a particular Pd compound for 30 min at 37 °C with shaking at 100 rpm was followed by DNA purification using a DNeasy Blood & Tissue Kit (Qiagen, Germany), and the DNA was subjected to qPCR. The identical procedure was performed for control samples with the exception that physiological saline solution was added instead of the Pd compound solution. Each condition was tested in biological duplicate. A schematic overview of the optimization of the treatment with Pd compounds is shown in Fig. 1.

### Initial optimization of palladium compound treatment conditions on *M. smegmatis* cells

Solutions of bis(benzonitrile)dichloropalladium(II) and palladium(II)acetate were added to the caps of the microtubes with 500 µl of viable and heat-killed cell suspensions to reach a 100 µM, 200 µM and 300 µM final concentration after mixing (Fig. 1). Physiological saline solution was added instead of the Pd compound solution in the case of control samples. The microtube caps were carefully closed, and the Pd-treated and untreated cell suspensions were uniformly spun and incubated at 37 °C with shaking at 100 rpm or 5 °C with manual shaking every 10 min for 5, 10, 15, 30 or 60 min depending on the concentration applied. The microtubes were centrifuged at 7,000 × g for 5 min, and the supernatant was removed. Then, either DNA extraction (see below) from the cell pellet was performed immediately or the cells were additionally washed with ultrapure water before extraction. Each condition was tested in biological duplicate.

### Application of the palladium compound treatments on MAP cells

After initial optimization on *M. smegmatis* cells, optimization of the viability assay continued on the target MAP cells. Live and dead MAP cell suspensions were treated with 30 µM (acting for 90 min), 50 µM (30 min) and 100 µM (15 min) bis(benzonitrile)dichloropalladium(II) and palladium(II)acetate at 5 °C (with manual shaking about every 10 min) followed by centrifugation at 7,000 × g for 5 min, washing with ultrapure water and DNA extraction (Fig. 1).

### Viability assay utilizing propidium monoazide

The procedure previously described in the study by Kralik et al.^8^ was followed for PMA treatment. In brief, 12.5 µl of PMA was added to 500 µl aliquots of live and dead MAP cells to achieve a final 25 µM concentration. The microtubes were briefly spun and incubated for 5 min at room temperature with shaking at 1,200 rpm under minimal light conditions. Subsequently, the microtubes placed horizontally on ice were irradiated with light (a halogen lamp with a 650 W bulb) for 2 min from a distance of 20 cm. This treatment with PMA was repeated once more, and the microtubes were then centrifuged at 7,000 × g for 5 min. The supernatant was removed, followed by DNA extraction from the cell pellets. The identical procedure was performed in the case of controls except that 20% DMSO was added instead of PMA. This experiment was carried out in biological duplicate.

### DNA extraction after treatment with palladium compounds and propidium monoazide

DNA was extracted as a crude lysate as described in the study by Kralik et al.^8^, in which this procedure showed comparable outcomes to DNA isolation kits. The cell pellets were resuspended in 500 µl of ultrapure water in the case of *M. smegmatis*. For MAP, only ultrapure water was used at the initial treatment with 50 and 100 µM Pd compounds, while ultrapure water and TE buffer supplemented with carrier DNA solution (salmon sperm DNA, 50 ng/µl, Serva, Germany), both in a volume of 500 µl, were compared as the media used to resuspend the cell pellets in the case of subsequent treatment with 30 µM. Only TE buffer with carrier DNA solution was used in the case of treatment with PMA. The resuspended cells were lysed in a thermoblock at 100 °C for 20 min, then centrifuged at 14,000 × g for 5 min, and the supernatant containing DNA was collected and analyzed by qPCR.

### Quantitative PCR, data analysis and statistics

qPCR assay targeted the ITS (internal transcribed spacer) sequence for *M. smegmatis*^15^ and the *F57* sequence for MAP^16^. An internal amplification control (IAC) was included in both qPCR assays. All samples were analyzed in technical duplicates using a LightCycler 480 (Roche Molecular Diagnostics, Germany).

The “Fit Point Analysis” of the LightCycler 480 software (version 1.5.0.39) was used to determine Cq values, from which the differences (ΔCq) between purified MAP DNA or live and dead mycobacterial cells treated with Pd compounds or PMA and the respective control (untreated) samples were calculated. The following formulas for calculating the ΔCq were, similarly to the previous study^14^, taken with slight modifications from the study by Kralik et al.^8^:

1 $$\Delta {\text{Cq}}\,_{{{\text{DNA}}\;{\text{with}}\;{\text{Pd}} {-} {\text{DNA}}\;{\text{without}}\;{\text{Pd}}}}$$

2 $$\Delta {\text{Cq}}\,_{{{\text{dead}}\;{\text{with}}\;{\text{Pd}}/{\text{PMA}}{-} {\text{live}}\;{\text{with}}\;{\text{Pd}}/{\text{PMA}}}}$$

3 $$\Delta {\text{Cq}}\,_{{{\text{dead}}\;{\text{with}}\;{\text{Pd}}/{\text{PMA}}{-} {\text{dead}}\;{\text{without}}\;{\text{Pd}}/{\text{PMA}}}}$$

4 $$\Delta {\text{Cq}}\,_{{{\text{live}}\;{\text{with}}\;{\text{Pd}}/{\text{PMA}}{-} {\text{live}}\;{\text{without}}\;{\text{Pd}}/{\text{PMA}}}}$$

Equation (1) describes the difference in amplification of the target sequence of Pd-treated and untreated MAP DNA samples. The highest possible ΔCq value is desirable, i.e. the greatest possible suppression of target sequence amplification by the Pd compound (without affecting the IAC). Equation (2) indicates the extent to which Pd or PMA suppresses target sequence amplification in dead cells, with the resulting value being reduced by the extent to which the agent undesirably suppresses amplification in live cells. Equation (3) also indicates the extent to which Pd or PMA suppresses amplification in dead cells, though regardless of live cells. For both Eqs. (2) and (3) the highest possible ΔCq value is desirable. Equation (4) indicates the extent to which Pd or PMA undesirably suppresses target sequence amplification in live cells, i.e. the lowest possible ΔCq value is required.

In the statistical analysis of ΔCq, one-way and two-way analysis of variance (ANOVA) and Tukey’s HSD test were applied using the statistical software Statistica 13.2 (StatSoft Inc., Tulsa, OK, USA). P-values less than 0.05 were considered statistically significant.

## Results

### The effect of palladium compounds on DNA amplification

**Figure 2:**
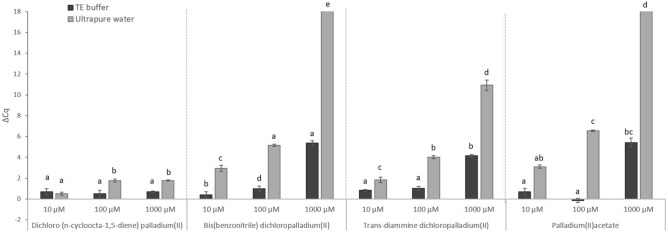
The effect of Pd compound solutions at three concentrations on the amplification of MAP DNA diluted with TE buffer or ultrapure water. The ΔCq values represent the mean difference of Cq values of Pd-treated and mean Cq value of Pd-untreated control samples counted from four biological and technical replicates. Two-way ANOVA with factors of concentration (10, 100, 1000 µM) and dilution medium (TE buffer, ultrapure water) and Tukey´s HSD test were used to evaluate the significance of differences between ΔCq, with all Pd compounds evaluated separately. Identical letters signify statistically insignificant differences (P > 0.05) between ΔCq values, and different letters signify significant differences (P < 0.05). Error bars express standard deviations counted from four replicates. The bars with ΔCq value above 18 indicate that no amplification occurred.

In the first step, the binding ability of four Pd compounds to purified MAP DNA was evaluated (Fig. 2). The ΔCq values of Pd-treated and untreated control samples were calculated according to Eq. (1). The highest suppression of amplification was found for bis(benzonitrile)dichloropalladium(II) and palladium(II)acetate, in which complete qPCR signal eliminations were achieved at a concentration of 1,000 µM. This experiment also examined the impact of two different media (TE buffer and ultrapure water) used to dilute the DNA on the ability of Pd compounds to chelate the target DNA sequence (Fig. 2). TE buffer proved to be an unsuitable dilution medium, as its utilization resulted in a significantly lower reduction in DNA amplification as compared to ultrapure water. Based on these results, bis(benzonitrile)dichloropalladium(II) and palladium(II)acetate were selected for subsequent optimization using live and heat-killed *M.* *smegmatis* and MAP cells diluted in ultrapure water.

### Initial optimization of palladium compound treatment conditions on *M. smegmatis* cells

**Figure 3:**
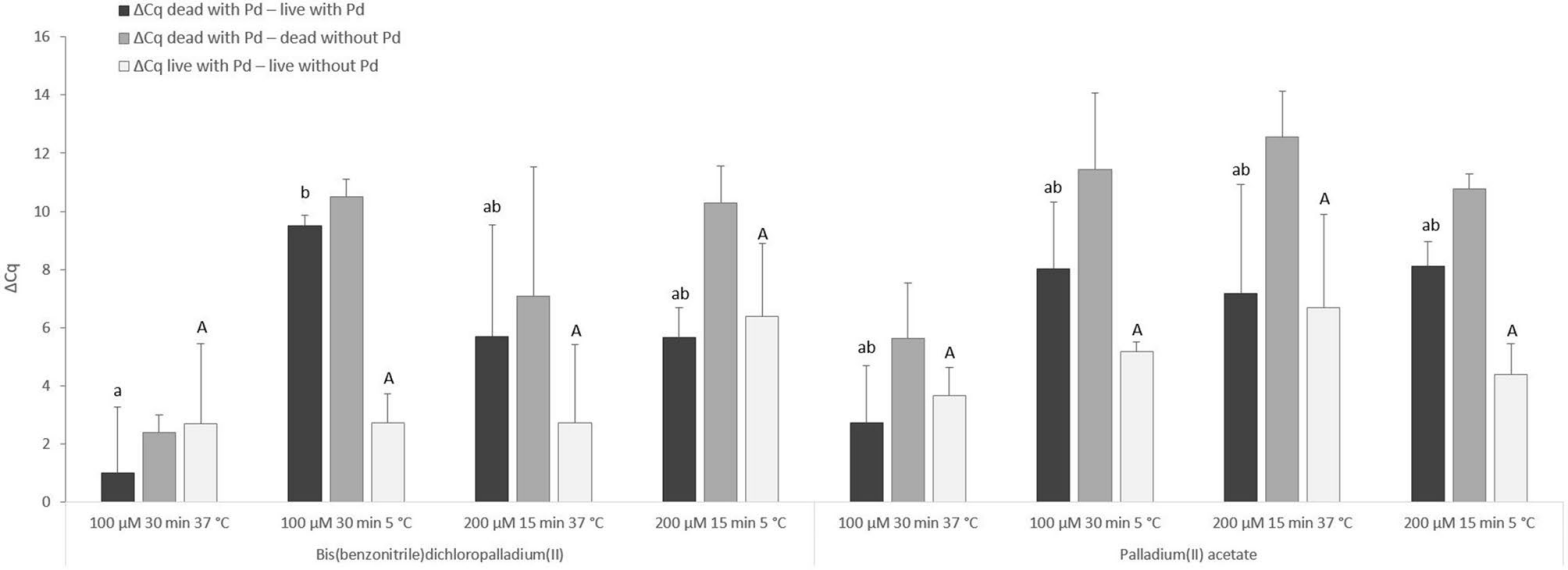
The effect of treatment of *M. smegmatis* cells with bis(benzonitrile)dichloropalladium(II) and palladium(II)acetate at two different concentrations (100 µM, 200 µM), times (30 min, 15 min) and temperatures (37 °C, 5 °C). The ΔCq values represent the individual mean differences of Cq values counted from four biological and technical replicates. One-way ANOVA and Tukey´s HSD test were used to evaluate the significance of differences between ΔCq. Identical letters signify statistically insignificant differences (P > 0.05) between ΔCq values, and different letters signify significant differences (P < 0.05). The values of ΔCq _dead with Pd – live with Pd_ were labeled with lowercase letters, and the values of ΔCq _live with Pd – live without Pd_ were labeled with capital letters, since they were assessed separately. Error bars express standard deviations counted from four replicates.

Bis(benzonitrile)dichloropalladium(II) and palladium(II)acetate at concentrations of 100 µM, 200 µM and 300 µM were used for the initial treatment of live and heat-killed cell suspensions at 37 °C. Since substantial inhibitions of IAC amplification occurred in qPCR, an additional washing step was added in the following experiment, in which only concentrations of 100 µM acting for 30 min and 200 µM for 15 min were applied (Fig. 3). In addition, the effect of temperature (37 °C and 5 °C) during the treatment with the two Pd compounds was investigated in this experiment. For both Pd compounds, a concentration of 100 µM acting for 30 min at 5 °C appeared to be the most suitable of the conditions tested (the highest values of ΔCq _dead with Pd – live with Pd_ and ΔCq _dead with Pd_ _– dead without Pd_), although a high level of undesirable penetration of both Pd compounds into live cells was observed (a value of ΔCq _live with Pd – live without Pd_ greater than 2 Cq). Thereafter, exposure to Pd compounds at a concentration of 100 µM was retested for 15 min, although at 5 °C with double washing. This did not, however, result in lower permeation of the Pd compounds into live cells (data not shown).

### Application of the palladium compound treatments on MAP cells

**Figure 4:**
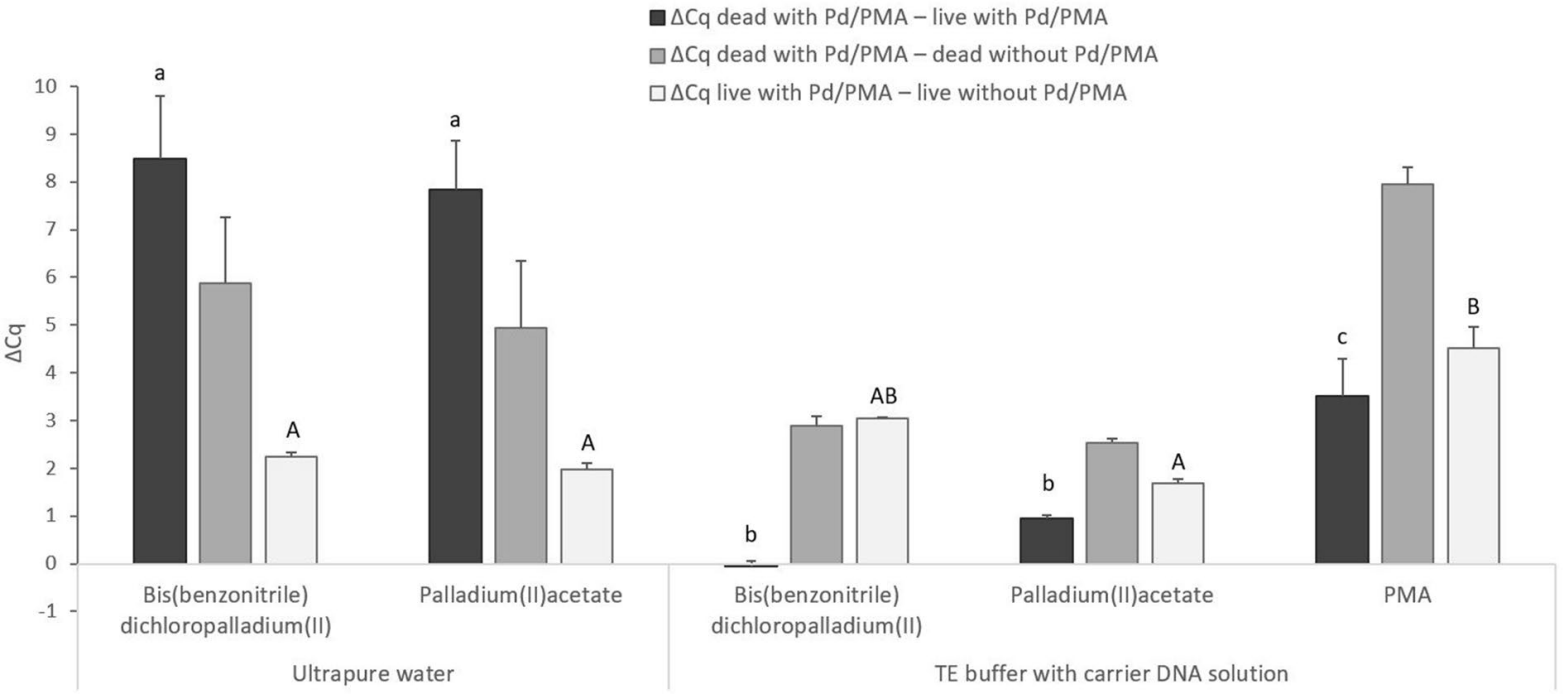
Comparison of treatment of MAP cells with bis(benzonitrile)dichloropalladium(II), palladium(II)acetate and PMA, and the effect of two different resuspension media (ultrapure water, TE buffer with carrier DNA solution) used after the Pd treatment. The ΔCq values represent the individual mean differences of Cq values counted from four biological and technical replicates. Two-way ANOVA with factors of agents used and resuspension medium and Tukey´s HSD test were used to evaluate the significance of differences between ΔCq. Identical letters signify statistically insignificant differences (P > 0.05) between ΔCq values, and different letters signify significant differences (P < 0.05). The values of ΔCq _dead with Pd/PMA – live with Pd/PMA_ were labeled with lowercase letters, and the values of ΔCq _live with Pd/PMA – live without Pd/PMA_ were labeled with capital letters, since they were assessed separately. Error bars express standard deviations counted from four replicates.

Since a high degree of permeation of both bis(benzonitrile)dichloropalladium(II) and palladium(II)acetate into live *M. smegmatis* cells was observed at a concentration of 100 µM, a lower concentration of 50 µM (30 min at 5 °C) was tested in addition to 100 µM (15 min at 5 °C) when applying the viability assay to MAP cells. However, a high value of ΔCq _live_ _with Pd – live without Pd_ of about 3 Cq was still recorded for both conditions (data not shown). The live and heat-killed MAP cells were subsequently exposed to an even lower concentration of 30 µM for an extended period of time (90 min) at 5 °C and compared to a reference viability assay using PMA. For Pd compounds, the effect of ultrapure water and TE buffer with carrier DNA solution used to resuspend cell pellets after the first wash was also evaluated (Fig. 4). When using ultrapure water, the viability assay utilizing bis(benzonitrile)dichloropalladium(II) and palladium(II)acetate attained differences in Cq values between treated live and dead MAP cells of 8.5 and 7.9, respectively, corresponding to approximately 2.5 log units. Regarding the permeation of Pd compounds into live MAP cells, values of ΔCq _live with Pd– live without Pd_ of slightly above and slightly below 2 Cq were recorded for bis(benzonitrile)dichloropalladium(II) and palladium(II)acetate, respectively. The second resuspension medium tested – TE buffer with carrier DNA solution – showed a significant drop in the ΔCq values of Pd-treated live and dead MAP cells below 1 Cq for both Pd compounds compared to ultrapure water. TE buffer with carrier DNA solution is therefore not a suitable medium in a viability assay using these Pd compounds. Nevertheless, it should be noted that ultrapure water is probably not a suitable medium either, as slightly reduced DNA amplification in the dead-cell control was recorded in MAP, though not detected in *M. smegmatis*. In the viability PCR using PMA, the difference in Cq values between PMA-treated live and dead MAP cells attained a value of about 3.5, which was significantly lower compared to both Pd compounds and, in addition, PMA penetrated more significantly into live MAP cells (Fig. 4).

## Discussion

Cultivation is currently a standard method for assessing MAP viability, but it is very time-consuming (up to several months). In contrast, viability PCR is capable of viability evaluation within one day. However, the currently used PMA-qPCR requires demanding procedures involving manipulation in a darkroom and on ice. The aim of this study was to optimize a viability assay utilizing Pd compounds in combination with qPCR for the selective detection of live mycobacterial cells, specifically MAP, whose procedure is less demanding compared to PMA-qPCR and also less costly than both PMA-qPCR and the recently introduced Pt-qPCR^10^.

Four Pd compounds were tested for their ability to chelate MAP DNA manifested in a decrease in DNA amplification (Fig. 2). Bis(benzonitrile)dichloropalladium(II) and palladium(II)acetate showed the greatest suppression of DNA amplification. The direct effect of these Pd compounds on DNA amplification was also evaluated in the study by Soejima and Iwatsuki^10^ using purified *Cronobacter sakazakii* DNA. The same Pd compounds did not show any effect on norovirus RNA in another study addressing the detection of infectious noroviruses^12^.

A significant difference between the use of TE buffer and ultrapure water was demonstrated when a comparison of the two DNA dilution media was made (Fig. 2). Higher ΔCq values between Pd-treated and untreated DNA samples were recorded when using ultrapure water as compared to TE buffer. The reason for the lower reduction in amplification in the case of TE buffer was probably the fact that the Pd compounds used in our study apparently formed chelates with the EDTA, as is confirmed by previous studies on Pd complexes^17,18^, and these were consequently unable to bind to DNA. Ultrapure water was therefore used to dilute mycobacterial cell suspensions in subsequent viability assays.

The treatment with the Pd compounds bis(benzonitrile)dichloropalladium(II) and palladium(II)acetate, which showed the greatest reduction in the qPCR signal when evaluated for the direct effect on MAP DNA, was then optimized on mycobacterial cells. Initial optimization steps were performed on *M. smegmatis*, as a model mycobacterium due to its short incubation time, with a continuation on the target slow-growing MAP. Based on the optimization procedures examining various concentrations, exposure times and temperatures, the optimal conditions for the Pd treatment of MAP cells were defined as 90 min exposure to a 30 µM concentration at 5 °C (Figs. 3 and 4). The differences noticed between Pd-treated live and dead MAP cells under these optimized conditions were 8.5 and 7.9 Cq for bis(benzonitrile)dichloropalladium(II) and palladium(II)acetate, respectively, i.e. about 2.5 log units. The results achieved in the present study confirmed the findings of the previous study by Soejima and Iwatsuki^10^ that Pd compounds are effective at much lower concentration than Pt compounds that have recently been applied to MAP^14^, which is important in terms of cost reduction. The maximum value of the difference between the Cq of Pt-treated live and dead MAP cells achieved in the above study was slightly above 6 Cq when treated with 100 µM *cis*-dichlorodiammine platinum(II), which is lower by about 2.5 Cq and 2 Cq compared to bis(benzonitrile)dichloropalladium(II) and palladium(II)acetate, respectively, at a concentration of 30 µM, as evaluated in the present study. Based on these findings, Pd compounds seems to be superior to Pt compounds in viability PCR applied to MAP. To date, the Pd compounds have been used to assess viability only in the enterobacteria *Escherichia coli* and *C. *s*akazakii*^10^, in which complete suppression of the qPCR signal of dead cells was achieved. In our study, absolute elimination of the qPCR signal was not attained for either dead MAP or *M. smegmatis* cells. This is probably caused by a more complex mycobacterial cell wall, through which Pd compounds are less permeable even in membrane-compromised dead cells. Another probable factor could be the use of a short amplicon (< 150 bp), which made it impossible to eliminate the qPCR signal more efficiently^9^. Furthermore, the efficient chelation of the Pd compound molecules with the target DNA could be reduced, as it has been suggested the Pd molecules possibly also adsorb to cell wall transmembrane proteins and DNA-binding proteins^10^. Regarding the undesirable impact of Pd compounds on live MAP cells, the differences observed between Pd-treated and untreated live MAP cells were around 2 Cq for both bis(benzonitrile)dichloropalladium(II) and palladium(II)acetate. Similarly, in a study by Soejima and Iwatsuki^10^, differences between Pd-treated and untreated live enterobacteria of up to 2.1 Cq were recorded, which was evaluated by the authors as an insignificant permeation. In addition to the possible limited penetration of Pd molecules into live cells, which, as in the case of dead cells, may result in suppression of target DNA amplification, the slight increase in Cq values for non-heat-treated cells after Pd-treatment could also be caused by the presence of membrane-compromised cells naturally occurring in the viable cell suspension. We tried to estimate the number of viable cells in the initial *M. smegmatis* suspension retrospectively by calculating CFU on plated serial dilutions. However, it greatly underestimated (by about 1.5–2 log units) the cell concentrations due to the clustering of mycobacteria as compared with the determination by qPCR (data not shown). These results are consistent with the study by Elguezabal et al.^7^, who evaluated that the qPCR of a single-copy gene is a more reliable method for determining mycobacterial numbers than cultivation, which we followed in our study for both *M. smegmatis* and MAP.

The final experiment also demonstrated the effect of the resuspension medium used after the first washing of the cells following the Pd-treatment on the viability assay (Fig. 4). Since heat-killed Pd-untreated MAP cells showed a decrease in the target sequence amplification when using ultrapure water as the resuspension medium, although not in *M. smegmatis*, the use of DNA-stabilizing and loss-reducing^16^ TE buffer with carrier DNA solution was also tested. Nevertheless, the use of TE buffer with carrier DNA solution resulted in a significantly lower difference in Cq values between Pd-treated live and dead MAP cells (below 1 Cq) compared to the use of ultrapure water. Consequently, the TE buffer should not be used in a viability assay either as a dilution or as a resuspension medium as it impairs the ability of Pd compounds to suppress amplification of the target sequence in dead cells. Decreases in DNA amplification of 1.5 Cq and 2.3 Cq in heat-killed Pd-untreated *C. sakazakii* and *E. coli* cells, respectively, compared to live Pd-untreated cells both diluted in water were also recorded in the study by Soejima and Iwatsuki^10^. It is therefore necessary to find a more suitable medium that does not distort the viability assay utilizing Pd compounds.

The efficiency of the viability assay Pd-qPCR was compared with PMA-qPCR (Fig. 4), which has previously been applied in the viability determination of MAP cells^8^. Both bis(benzonitrile)dichloropalladium(II) and palladium(II)acetate (in the case of the use of ultrapure water as the resuspension medium) allowed higher differences in Cq values between Pd-treated live and dead MAP cells, by about 5 Cq and 4.3 Cq, respectively, than PMA dye. In addition, PMA showed higher permeability into live MAP cells than Pd compounds. Pd compounds thus proved to be a more suitable viability marker than PMA. Likewise, Soejima and Iwatsuki^10^ evaluated Pd compounds as superior to the PMA agent for distinguishing between live and dead enterobacteria in water.

In summary, our study optimized a viability assay combining Pd compound treatment with qPCR to detect live MAP cells diluted in ultrapure water. Pd-qPCR proved to be more effective in distinguishing between live and dead MAP cells as compared to the previously established PMA-qPCR. In terms of practical use, further studies could focus on evaluating the ability of the optimized protocol to detect viable MAP cells in milk, tissue or feces from infected animals or environmental samples. If efficacy is demonstrated in clinical samples, Pd-qPCR will facilitate the identification of MAP-infected cattle, thus ensuring effective monitoring of paratuberculosis.
